# Case of Injury of the Brain, Occasioned without Any Blow or External Violence upon the Head

**Published:** 1784

**Authors:** 


### III. *Case of Injury of the Brain, occasioned without any blow or external violence upon the Head. Communicated in a letter to Dr.* Simmons, F.R.S. *by Mr.* William Houlston, *Member of the Corporation of Surgeons, London.*

A LADY of Salisbury Street in the Strand, was walking out during the frost in February last, and in consequence of treading upon a part of the pavement, rendered slippery by the frequent passing of persons in the street, received a smart fall. As she fell in a sitting posture, her feet flying suddenly from under her, she seemed to have sustained no material hurt, and being assisted in rising by some of the by-standers, readily walked home. On the evening of the following day a pain in her head came on, and she felt some inclination to be sick. She passed the night without sleep, and on the third day from the time of the accident complained greatly of a kind of sensation within her head unlike any thing she had ever before experienced. It was, to use her own words, a drowsy, heavy pain, and giddiness; with dimness of sight, and a sort of weight in the forehead, which when she leaned forward forward ſeemed to incline her head towards the ground. She had not as yet any vomiting, nor was there any obſervable alteration in the pulſe. Upon being deſired to point out the particular ſituation of the pain ſhe complained of, ſhe ſaid it was not fixed to any diſtinct part, except when her head had remained long in one poſture, and then that ſide which preſſed againſt the pillow was the moſt painful, and became ſore to the touch. Although the head in this caſe had not received the leaſt ſtroke or outward violence, theſe were evidently the ſymptoms of approaching miſchief, perhaps from concuſſion, and I, therefore, though in vain, deſired her to loſe ſome blood. She had unfortunately ſuch a dread of the lancet, as to have determined never, in any inſtance, whatever might be the conſequence, to ſubmit to its uſe; I, therefore, directed an opening medicine, a cooling regimen, and the avoiding every thing whieh might diſturb the mind, or act powerfully on the external ſenſes. The next day ſhe found herſelf better, and getting up, remained the whole day with the family, though greatly incommoded by the ſmalleſt noiſe, and obliged to recline upon a pillow, and ſometimes upon a ſofa. The pain in her head, though not without fome intermiffion, was now exceedingly violent, and in the evening fhe began to vomit, and her pulfe was fuller and more frequent than natural, till at length finding herfelf grow worfe, fhe very willingly retired to bed. She was ordered laxative clyfters, and the ufe of the pediluvium ; and as I thought the cafe required that fomething effectual fhould be attempted, I directed the ufe of what Mr. Bromfeild in his firft volume of Obfervations calls the *fudorific anodyne*, confifting of one drachm of Thebaic tincture and three drachms of antimonial wine. Of this twenty drops were given in a cupful of barley water. On the fifth day, having flept a good deal, though with fome interruption, fhe was better, and got up, complaining that the forenefs of her head increafed by her continuing in bed. Towards afternoon fhe became worfe, from being fatigued with too much company, had frequent returns of ficknefs, and at night vomited, as fhe faid, a teacup-full of blood. The anodyne draught was repeated, together with the methods before recommended. I preffed her to lofe blood from the jugular veins, but even the application of leeches was not to be admitted. Soon after taking the

 medimedicine, a ſevere rigor took place, ſucceeded by a hot fit of two hours, and a ſlight delirium; but as the morning approached, a mild ſweat came on, and produced great relief of all the ſymptoms. She was kept in bed all the ſixth day, and took ten of the anodyne drops every three hours; but as they rather produced a hot and dry than a relaxed ſkin, the form of the medicine was altered on the ſeventh to a ſolution of emetic tartar, three grains in five ounces of cinnamon water, with ſixty drops of Thebaic tincture, two drachms of nitre, and an ounce of common ſyrup. Of this ſhe took a table ſpoonful every three hours, paſſed the day better than ſhe had hitherto done, had leſs pain in her head, and kept on her ſtomach a light dinner of bread pudding. In the evening ſhe began to perſpire, and continued in that ſtate all the night. The eighth, ninth, and tenth days ſhe continued to mend, though both her head and ſtomach were occaſionally affected, and the light was particularly offenſive to her eyes, though I could never, upon examination, diſcover any dilatation of the pupil that could be deemed morbid. On the twelfth day, notwithſtanding theſe favourable appearances, ſhe experienced a relapſe, had a very ſevere rigor in the evening, and a return of all the ſymp- toms. This, however, I found was accounted for by ſome little irregularity in her diet, and the omiſſion of her medicine, which I ordered to be repeated; and by the continuance of which, with the occaſional uſe of laxatives, ſhe gradually, but perfectly recovered.

I confeſs, that in this caſe, if bleeding had been permitted, I ſhould not have had recourſe to the anodyne treatment. It appears, how- ever, from the good effects produced here, that ſudorific remedies of this kind are by no means to be held in a ſecondary light; and we may farther obſerve, that in conſtitutions particu- larly affected by opium, where the ſtomach yields more to the anodyne than to the diapho- retic power of the drops adviſed by Mr. Brom- feild, and where on that account their ſudorific effect is not produced, but, on the contrary, a dry and hot ſkin; in that caſe, it ſhould ſeem moſt adviſeable ſo to augment the quantity of antimonial wine as to obviate this objection. Though there may be a tendency to vomiting, an increaſe of this diſpoſition certainly need not be apprehended; for, till a certain degree of this nauſea *is* induced, ſuch a degree, at leaſt, as is juſt within the power of the opiate to overovercome, a ſweat, we are told, cannot be produced. This ſeems to be one of thoſe caſes to which Mr. Pott alludes when ſpeaking of injury of the brain, he ſays, "It may be produced, not only when the cranium is unhurt "by the blow, but even when no violence of "any kind has been offered to, or received by "the head." A very eminent gentleman of the profeſſion lately told me of a boy who died of an affection of the brain, occaſioned by a ſtroke on the eye by the laſh of a whip; and certain it is, that the head is liable to very ſerious injuries from cauſes not altogether violent.

*Palſgrave Place,*

*Auguſt* 26, 1784.

